# Comprehensive tuberculosis screening and preventive treatment in schools and congregate settings of India (2017–2024): a prospective study

**DOI:** 10.1016/j.lansea.2026.100725

**Published:** 2026-02-06

**Authors:** Kunchok Dorjee, Sonam Topgyal, Rajesh K. Sood, Tenzin Namdon, Ravinder Kumar, Ugen Gyatso, Jigme Kalsang, Tenzin Thinley, Tenzin Dechen, Tenzin Tsomo, Tenzin Kalsang, Rachel C. Sadoff, Sangyal Dorjee, Sheriza Baksh, Tenzin Yangkyi, Tenzin Khachoe, Tenzin Dolker, Tsering Wangmo, Dekyi Lhadon, Lobsang Tsering, Amita Gupta, Robert C. Bollinger, Jonathan E. Golub, Vidya Mave, Sourya Shrestha, Zorba Paster, Richard E. Chaisson, Tsetan D. Sadutshang

**Affiliations:** a Division of Infectious Diseases, Johns Hopkins University School of Medicine, Baltimore, MD, 21205, USA; b Delek Hospital, Department of Health, Central Tibetan Administration, Dharamshala, Himachal Pradesh, 176215, India; c Department of International Health, Bloomberg School of Public Health, Baltimore, MD, 21205, USA; d Gupta-Klinsky India Institute, Johns Hopkins University, Baltimore, MD, 21287, USA; e National TB Elimination Program, National Health Mission, Shimla, Himachal Pradesh, 171009, India; f Department of Epidemiology, Bloomberg School of Public Health, Baltimore, MD, 21205, USA; g Department of Health, Central Tibetan Administration, Dharamshala, Himachal Pradesh, 176219, India; h Centre for Infectious Diseases in India, Johns Hopkins India, Pune, Maharashtra, 411001, India; i Department of Family Medicine, University of Wisconsin–Madison, Madison, WI, 53705, USA

**Keywords:** Tuberculosis, Children or adolescent, Congregate settings, Preventive treatment, TB transmission, TB elimination

## Abstract

**Background:**

Existing Tuberculosis (TB) elimination strategies show limited impact, with suboptimal uptake of tuberculosis preventive treatment (TPT) and increasing TB incidence after the COVID-19 pandemic. Real-world evidence on reduction of tuberculosis in high-burden communities is needed to inform future TB elimination strategies.

**Methods:**

Since 2017, a comprehensive TB screening and TPT program known as Zero TB in Kids (ZTBK) was implemented in congregate settings of Tibetan communities in India. TB disease, TB infection (TBI), tuberculin skin test (TST) conversion, and TPT uptake were measured periodically.

**Findings:**

Schoolchildren and adults in 63 institutes (n = 20,068; 67,637 person-years) were screened. TPT was given to 3847 participants. TB incidence decreased 83% between 2017 [576 (95% CI: 455–718)/100,000] and 2024 [97 (47–179)/100,000]. TB infection (TBI) prevalence decreased 32% between 2017 [22% (95% CI: 21–23%)] and 2024 [15.5% (14–17%)]. TB incidence (640/100,000) and TBI prevalence (28%) were higher in the institutes that were never screened before under ZTBK. Among participants who did not receive TPT, TB disease prevalence decreased 84% between 2017 [910 (95% CI: 675–1204)/100,000] and 2024 [147 (48–343)/100,000], indicating a herd benefit. After one round of TB screening and TPT, between 2018 and 2019, TST conversion decreased 59% for children and 47% for adolescents. Risk of TBI was greater for males (aPR: 1.23; 95% CI: 1.16–1.30). TB risk was 82% lower for schoolchildren receiving TPT. Participants with seizure disorder [aPR: 0.31 (95% CI: 0.15–0.65)] and hepatitis B [0.71 (0.6–0.84)] were less likely to receive TPT.

**Interpretation:**

Significant reduction of TB transmission and burden can be achieved using the existing tools of TB control. Surveillance of TBI and TPT must be widely adopted for schools and congregate settings with high TB burden.

**Funding:**

10.13039/100000002National Institutes of Health-10.13039/100000060National Institute of Allergy and Infectious Diseases (NIAID) (K01-AI148583), STOP TB Partnership (STBP/TBREACH/GSA/W7-7692), NIAID-Johns Hopkins Center for AIDS Research (90100777), Foundations, and Philanthropy.

**Research in context**

**Evidence before this study**

We searched PubMed for published studies between January 2000 and December 2025 using the mesh terms (“Tuberculosis”) AND (“Mass Screening” OR “Antibiotic Prophylaxis” OR “Isoniazid”) AND (“Incidence” OR “Prevalence”). The search yielded 301 articles. We then reviewed title and abstract to identify studies describing community or population level effectiveness of TB screening or preventive treatment on reduction of TB burden. We identified 23 relevant articles. Most of these studies were in communities with high HIV prevalence or were evaluation studies among TB contacts. In communities with low HIV and high TB prevalence, which constitutes majority of the global TB burden, we could not identify any study that demonstrated meaningful reduction of TB, except for one study we had published to show the impact on TB reduction over three years. This study addresses significant gaps in knowledge and experience regarding the effects of large-scale TB screening and tuberculosis preventive treatment (TPT) globally and in Southeast Asia.

**Added value of this study**

In a prospective cohort of schools, monasteries and nunneries, comprehensive and longitudinal TB screening and TPT implemented over eight years between 2017 and 2024 led to 83% reduction of TB disease incidence and 32% reduction of TBI prevalence, despite the setbacks from the COVID-19 pandemic. Prevalence of TB disease decreased substantially among participants who did not receive TPT also, indicating a population level or herd benefit of TPT. After one round of screening and TPT, TST conversion declined 59% in children and 47% in adolescents in one year. The pandemic had led to resurgence of TBI, TST conversion, and TB disease that however declined again in 2024 after continued screening and TPT. Participants with seizures and hepatitis B were less likely to receive TPT. Reason were likely physicians’ uncertainty, lack of confidence, or cautiousness in prescribing TPT due to fear of hepatotoxicity or drug interactions. Our findings of increased risk of TBI and disease in children and adolescents recently exposed to TB imply ongoing transmission as a driver of TB burden in this population, underscoring the importance of screening and TPT.

**Implications of all the available evidence**

Rapid and sustained reduction of TB transmission and TB burden in high TB burden communities is possible with the optimized use of existing tools of TB control.

Guidelines are needed to implement TPT in people with comorbidities such as hepatitis B and seizures for improved uptake and acceptance of TPT. A shift in the approach of global TB control to emphasize surveillance of TBI and TPT implementation is needed. This study provides a real-world framework for ending TB, outside of mathematical models.

## Introduction

Tuberculosis (TB) continues to pose a major global health challenge. An estimated 11 million people are affected annually by TB with 1.3 million deaths, making it the leading infectious cause of mortality.^1^ 12% of the TB disease and 15% of TB deaths occur in children under 15 years of age. The shortcomings of the existing TB control approach, which is centered on disease detection are evident in the modest 2% average annual decline of TB incidence and a rise of TB incidence since the COVID-19 pandemic. Additionally, there is a lack of data on the annual prevalence of TB infection (TBI), primarily due to the absence of a standardized system for surveillance of TBI. Despite a proven efficacy of tuberculosis preventive treatment (TPT), its implementation and uptake remain suboptimal.^1^ A major shift in approach is needed to encompass the detection of TBI and the prevention of transmission through a nuanced understanding and effective operationalization of TPT.

Studies showing population level reduction of TB from TPT implementation are largely lacking except for the early studies carried out in the Western hemisphere in the late 20th century when TB was prevalent there.^2^, ^3^, ^4^ Studies conducted in the 21st century on TPT were mostly focused in communities of high HIV prevalence or were hospital based studies evaluating effectiveness in contacts or specific risk groups. Contemporary evidence of population-level effectiveness of TPT are essentially lacking, more so in communities with low HIV and high TB prevalence, which constitutes most of the global burden of TB. Since 2017, we have implemented a comprehensive TB screening and preventive treatment program known as Zero TB in Kids (ZTBK) in congregate settings of the Tibetan communities in India, which had historically experienced high rates of TB disease.^5^^,^^6^ We have previously reported a high prevalence of TBI and disease in boarding schools^5^ and a significant reduction of TB after three years of ZTBK implementation.^7^ We observed that schoolchildren who had received TPT were 79% less likely to develop TB disease and that 4 months of rifampin (4R) was better tolerated by older children and adolescents as compared to 3 months of isoniazid and rifampin (3HR). The objectives of this study are to determine the long-term impact of the program on reduction of TB infection and disease in an expanded cohort. We describe the resilience of the program to unprecedented events such as the pandemic, as well as population level or herd benefits of TPT. Additionally, we characterize factors associated with TB infection and tuberculin skin test conversion; assess uptake, tolerance and completion of the TPT regimens; and discuss operational challenges of TPT implementation.

## Methods

The Tibetan community in the congregate settings of boarding schools, monasteries, and nunneries had disproportionately higher rates of TB.^5^^,^^8^ Consequently, in 2017, ZTBK was initiated as a collaborative effort of Delek Hospital and Johns Hopkins Center for TB Research under the Central Tibetan Administration Department of Health (CTADOH) and the National TB Elimination Program (NTEP) of the National Health Mission of Government of India. Although the institutes in the community annually received active case finding (ACF) supported by the NTEP, the ACF was focused on detecting active TB. Comprehensive screening for latent TBI and TPT implementation were never carried out prior to the initiation of ZTBK. Under ZTBK, students and adult staff members in schools, monasteries, and nunneries in the Tibetan community in the states of Himachal Pradesh and Uttarakhand were screened for TBI and disease using a mobile service delivery model. The institutes were mostly residential, with facilities such as dormitories, hostels, and staff-quarters. However, there were also day students who returned to their homes after school. Schoolchildren belonged to nearby as well as far-off high-altitude communities in the Himalayas. Throughout 2020 and 2021, TB screening could not be carried out due to the pandemic.

### Study design

All the congregate facilities in the two states were targeted for screening. Institutes were screened multiple times between 2017 and 2024. The frequency of the ZTBK screening depended on logistical feasibility and outbreak reports. The institutes constituted an open cohort with entry through annual enrollment of students and exit through graduation. Participants of all ages were included. Most institutes received at least one screening before the COVID-19 pandemic. Approximately one-third of the institutes were screened for the first time after the pandemic, serving as control for comparison with the institutes that received screening and TPT prior to the pandemic.

**Fig. 1:**
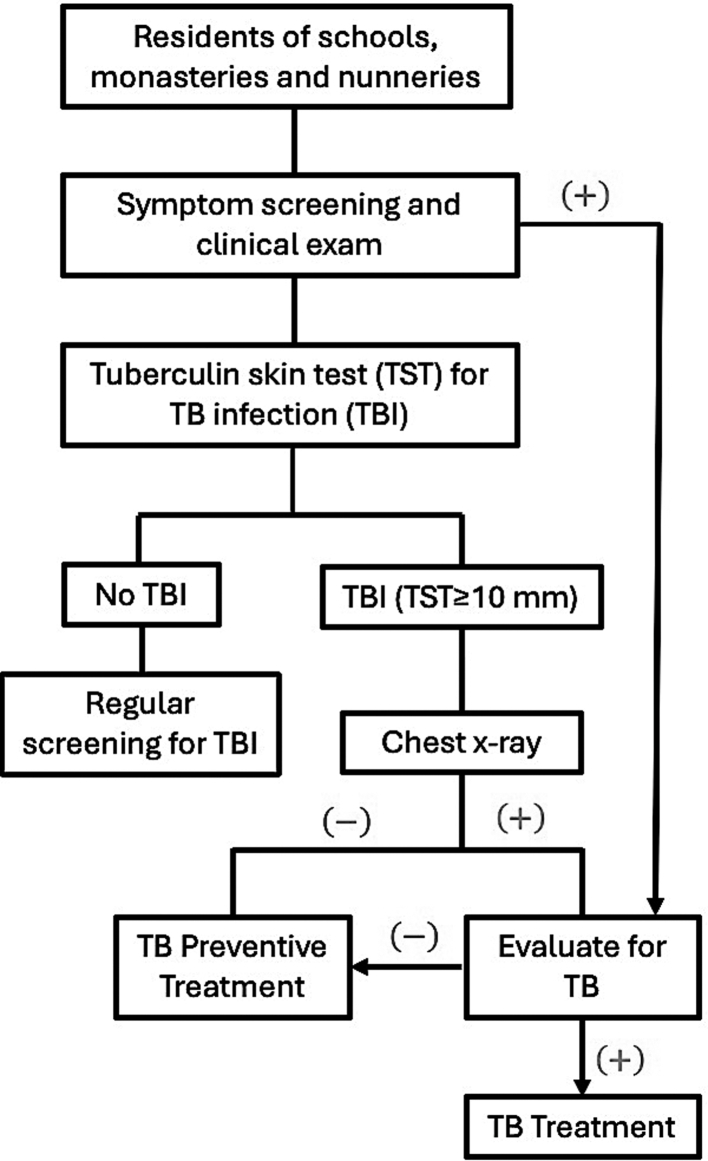
**Algorithm for TB screening and preventive treatment**.

Self-reported data on comorbidities including asthma, hypertension, diabetes, seizure, and hepatitis B were collected during in-person interviews. For participants under 18 years of age, information on co-existing conditions was provided by school nurses based on students’ personal health records. TB screening and TPT were implemented using a pre-defined algorithm (Fig. 1). TBI was determined by tuberculin skin testing consisting of intracutaneous injection of 5 TU of purified protein derivative RT23 (Span Diagnostics, Surat, India) in the forearm. Induration measuring ≥10 mm after 2–3 days was considered positive. Participants who had TBI or disease in the past did not repeat TST. Participants who were previously TST-negative were administered TST again at the subsequent screening to determine TBI acquisition in the interim, a measure of TB transmission. Active TB was confirmed using Xpert MTB/RIF assay or culture and treated under the national program. Gastric aspiration was performed for individuals unable to produce sputum. Initially, in 2017–2018, the TPT regimen consisting of three months of daily isoniazid and rifampicin (3HR) was used. From 2019 onward, four months of daily rifampicin (4R) was used due to reports of side-effects among older children such as feeling sleepy and tired during class.

A TB contact was defined if a child or staff member had shared the same dormitory or classroom with the index patient with TB disease in the past two years. The school clinics kept records of the TB affected individuals in schools. In addition, students were asked during screening if they have had contact with a TB affected individual at school or at home during vacation in the past two years. Further details on the program's initiation, sociocultural settings, and monitoring have been previously described.^5^^,^^7^

### Statistical analysis

Data were collected in-person with paper forms which were then entered into an electronic database.^5^^,^^7^ A unique ID was issued to each participant, enabling longitudinal analysis. Prevalence of TBI in an institute was calculated as the number of TST-positive persons out of all people receiving TST in the given year. Prevalence of TST conversion was calculated as the number of TST-negative persons in the previous screening converting to TST-positive in the given year. Log-binomial regression models were used to calculate prevalence ratios for the risk of TBI and TST conversion, accounting for clustering by institute. Log-Poisson model was used where the log-binomial model failed to converge. The models of TB infection risk or TST conversion were adjusted for age, sex, calendar year, residential facility, and TB exposure to provide adjusted prevalence ratio (aPR) estimates. TB incidence was the total number of new disease occurring among all residents of the institutes, including those detected actively during screening and those occurring passively. Denominator for the incidence was the total number of students and staff enrolled in the institutes. TPT effectiveness was determined by comparing schoolchildren who did and did not receive TPT using Cox proportional hazard regression to estimate adjusted hazard ratio (aHR). Participants were censored at the earliest of the first development of TB disease, graduation from the institute, or leaving the institute, or were otherwise administratively censored on December 31, 2024. Factors associated with TBI and TPT uptake were similarly assessed using Log-binomial regression. TPT regimen side-effects were compared using Chi-squared analysis. To measure reduction of TB transmission among participants who did not receive TPT, we calculated a population level effect or herd effect, referred to as herd effect henceforth, by measuring the change in prevalence of TB disease in participants who did not receive TPT.

### Ethics statement

The project was approved by the Johns Hopkins University School of Medicine Institutional Review Board (IRB00312350) and Delek Hospital Institutional Ethics Committee (#DLKIEC009043). IRB waived the informed consent requirement of this project as it was a public health initiative with no more than minimal risk to the participants. Oral permission was sought and received from parents or guardians for all children before administering TST or providing TPT, as well as from adult participants and school administrations. The project was endorsed by the CTADOH and Department of Education that administer the institutes. Two orientation sessions were held for the participants and parents/guardians at the respective institutes. Detailed information about the program was presented during the sessions, including the opportunity for the community to ask questions. Participants had the opportunity to refuse TB screening or TPT.

### Role of the funding source

The funders had no role in the collection, analysis, or interpretation of data; writing of the results; or decision to submit for publication.

## Results

**Fig. 2:**
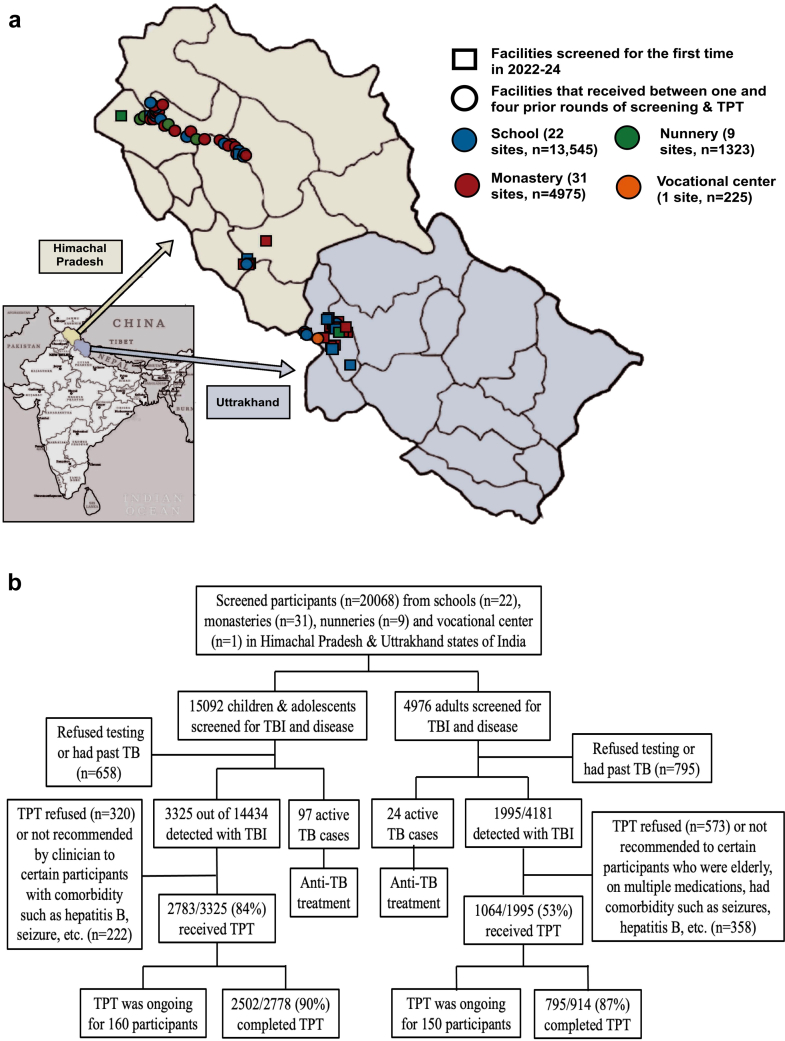
**Overview of TB screening and preventive treatment carried out in congregate settings in India.** a) Figure shows institutes that were screened under Zero TB in Kids (ZTBK) in the states of Himachal Pradesh and Uttrakhand, India between 2017 and 2024). b) Flow of participants in the study including participants screened for latent and active TB, detection of latent and active TB, and uptake and completion of TB preventive treatment.

Between January 2017 and December 2024, 63 institutes comprising 22 schools, 31 monasteries, 9 nunneries, and one vocational center were screened, enrolling 20,068 individuals who contributed 67,637 person years of follow-up time (Fig. 2A and B, Table 1). Most participants (68%) were from schools, followed by monasteries (25%), nunneries (7%), and a vocational center (1%). There were 9965 children aged 5–14 years, 5127 adolescents aged 15–19 years, and 4976 adults aged ≥20 years. Baseline characteristics are summarized in Table 1. Prevalence of past TB disease was 11.5% for adults and 1–3% for children and adolescents. Almost all participants (98.7%) had received the BCG vaccine. Prevalence of hepatitis B was 1% for children, 2% for adolescents, and 5% for adults.

**Table 1:** Baseline characteristics of children, adolescents, and adults screened for tuberculosis in the schools, monasteries and nunneries in Tibetan community in India (2017–2024).

Characteristics	All participants N = 20,068, n (%)	Children (5–14 years) N = 9965, n (%)	Adolescents (10–19 years) N = 11,793, n (%)	Adults (≥18 years) N = 6392, n (%)
Age, median (IQR)	15 (11–19)	11 (9–13)	14 (12–16)	28 (20–38)
Males	11,859 (59.1)	5708 (57.3)	6931 (58.8)	3995 (62.5)
Females	8209 (40.9)	4257 (42.7)	4862 (41.2)	2397 (37.5)
Weight (kg), mean (SD)	48.2 (17.5)	35.4 (11.4)	47 (12.7)	64 (13)
**Year of enrollment**				
2017	6177 (30.8)	3582 (35.9)	4162 (35.3)	1249 (19.5)
2018	6365 (31.7)	2269 (22.8)	3371 (28.6)	2914 (45.6)
2019	899 (4.5)	591 (5.9)	471 (4.0)	150 (2.4)
2022	1786 (8.9)	996 (10.0)	943 (8.0)	559 (8.8)
2023	3348 (16.7)	1517 (15.2)	1999 (17.0)	1196 (18.7)
2024	1493 (7.4)	1010 (10.1)	847 (7.2)	324 (5.1)
Follow up years for a person, mean (SD)	4.4 (2.8)	4.7 (2.8)	4.5 (2.7)	4.1 (2.7)
Total follow up time, person-years	67,637	31,493	40,535	23,093
**Institute**				
School	13,545 (67.5)	8313 (83.4)	9065 (76.9)	2407 (37.7)
Monastery	4975 (24.8)	1401 (14.1)	2306 (19.6)	2798 (43.8)
Nunnery	1323 (6.6)	250 (2.5)	373 (3.2)	968 (15.1)
Vocational center	225 (1.1)	0 (0)	49 (0.4)	219 (3.4)
**Place of birth (N = 19,249)**				
India	14,155 (70.6)	8155 (81.9)	8842 (75.0)	3338 (52.2)
Tibet	2228 (11.1)	161 (1.6)	647 (5.5)	1803 (28.2)
Nepal	3036 (15.2)	1494 (15.0)	2076 (17.6)	841 (13.2)
Bhutan	520 (2.6)	115 (1.2)	169 (1.4)	345 (5.4)
Other	67 (0.3)	24 (0.3)	57 (0.5)	63 (1.0)
**Ethnicity**				
Tibetan or Tibetan origin	9139 (91.7)	4613 (90.4)	10,665 (90.6)	5938 (93.2)
Indian or another ethnicity	823 (8.3)	490 (9.6)	1101 (9.4)	431 (6.8)
**Co-existing conditions (N = 19,296)**				
Ever smoked cigarette	286 (1.4)	–	152 (1.3)	181 (2.8)
Chronic lung disease	33 (0.2)	14 (0.1)	19 (0.2)	13 (0.2)
Hypertension	214 (1.1)	5 (0.05)	10 (0.1)	207 (3.2)
Diabetes mellitus	84 (0.4)	2 (0.02)	3 (0.03)	81 (1.3)
Hepatitis B	461 (2.3)	79 (0.8)	197 (1.7)	311 (4.9)
**Previous history of TB (N = 19,281)**				
Any TB in the past	989 (4.9)	129 (1.3)	310 (2.6)	733 (11.5)
Drug-susceptible TB (among past TB)	844 (85.3)	103 (79.8)	253 (81.6)	635 (86.6)
Drug-resistant TB (among past TB)	32 (3.2)	7 (5.4)	10 (3.2)	22 (3.0)
DST unknown (among past TB)	113 (11.4)	19 (14.7)	47 (15.2)	76 (10.4)
**TB contact in past 2 years**				
Any contact with TB case	3388 (16.9)	1244 (12.5)	2623 (22.3)	954 (14.9)
Contact with TB occurred at institute	3043 (15.2)	1051 (10.5)	2401 (20.4)	868 (13.6)
Contact with TB occurred at home	811 (4.0)	395 (4.0)	625 (5.3)	178 (2.8)
Contact with TB case among males	1685/11,852 (14.2)	597/5703 (10.5)	1253/6927 (18.1)	523/3993 (13.1)
Contact with TB case among females	1703/8202 (20.8)	647/4255 (15.2)	1370/4856 (28.2)	431/2396 (18.0)
**Received BCG vaccine (N = 19,727)**	19,644/19,910 (98.7)	9924/9940 (99.8)	11,623/11,765 (98.8)	6098/6271 (97.2)

None.

### Factors associated with TBI

Across the age-groups, the prevalence of TBI increased with age (Table 2). Males had a greater risk of TBI [aPR: 1.23; 95% CI: 1.16–1.30] and TST conversion [aPR: 1.34; 95% CI: 1.18–1.53] (Table 3, Fig. S1). Children with hepatitis B had higher risk of TBI [aPR: 1.99 (95% CI: 1.30–3.0) and a greater proportion of TST conversion. Recent exposure to TB was associated with greater risk of TBI in children [aPR: 1.39 (95% CI: 1.13–1.71] and adolescents [aPR: 1.45 (95% CI: 1.26–1.66)].

**Table 2:** Baseline characteristics and factors associated with TB infection in congregate settings in the Tibetan community in India (2017–2024).

Risk factor	All participants		Children (5–14 years)		Adolescents (10–19 years)		Adults (≥18 years)	
	TBI^a^ (N = 18,631), n (%)	aPR^b^ (95% CI); p value	TBI^a^ (N = 9681), n (%)	aPR^b^ (95% CI); p value	TBI^a^ (11,172), n (%)	aPR^b^ (95% CI); p value	TBI^a^ (N = 5495), n (%)	aPR^b^ (95% CI); p value
Age, median (IQR)	17 (13–28)	1.025 (1.02–1.03); <0.001	12 (10–13)	1.10 (1.05–1.15); <0.001	15 (13–17)	1.05 (1.02–1.08); 0.001	29 (21–40)	1.02 (1.01–1.02); <0.001
Males	2709/10,960 (24.7)	1.23 (1.16–1.30); <0.001	777/5523 (14)	1.18 (1.10–1.27); <0.001	1370/6544 (20.9)	1.31 (1.22–1.41); <0.001	1418/3430 (41.3)	1.21 (1.11–1.31); <0.001
Female	1538/7621 (20.2)	Reference	494/4128 (12)	Reference	785/4604 (17.0)	Reference	759/2049 (37.0)	Reference
Weight (kg), mean (SD)	54.7 (16.6)	1.01 (1.007–1.012); <0.001	39.2 (12.0)	1.0 (0.99–1.01); 0.63	49.4 (12.8)	1.00 (0.99–1.01); 0.15	65.5 (12.9)	1.01 (1.002–1.008); 0.003
**Year of screening**^c^								
2017	1247/5657 (22.0)	Reference	434/3337 (13.0)	Reference	817/3952 (20.7)	Reference	453/1038 (43.6)	Reference
2018	1607/6235 (25.8)	0.88 (0.75–1.04); 0.13	325/2332 (13.9)	1.08 (0.80–1.46); 0.61	698/3518 (19.8)	0.88 (0.70–1.09); 0.25	1007/2622 (38.4)	0.81 (0.68–0.97); 0.02
2019	452/3757 (12.0)	0.62 (0.56–0.70); <0.001	251/2525 (9.9)	0.77 (0.58–1.02); 0.07	315/2863 (11.0)	0.56 (0.47–0.68); <0.001	103/390 (26.4)	0.66 (0.50–0.88); 0.005
2022	577/3152 (18.3)	0.79 (0.64–1.98); 0.03	240/1749 (13.7)	1.07 (0.82–1.40); 0.60	340/2292 (14.8)	0.76 (0.61–0.94); 0.01	239/636 (37.6)	0.81 (0.58–1.12); 0.20
2023	552/3089 (17.9)	0.67 (0.55–0.81); <0.001	169/1401 (12.8)	0.82 (0.62–1.09); 0.17	291/2187 (13.3)	0.61 (0.47–0.79); <0.001	286/916 (31.2)	0.68 (0.55–0.86); 0.001
2024	481/3110 (15.5)	0.68 (0.50–0.93); 0.01	216/1693 (12.8)	1.03 (0.61–1.73) 0.91	296/2191 (13.5)	0.69 (0.48–1.01); 0.055	149/679 (21.9)	0.48 (0.36–0.63); <0.001
**Residential institute**								
School	2478/12,615 (19.6)	Reference	1034/8012 (12.9)	Reference	1630/8486 (19.2)	Reference	825/2015 (40.9)	Reference
Monastery/nunnery	1697/5776 (29.4)	1.20 (1.05–1.36); 0.007	236/1638 (14.4)	0.12 (0.93–1.36); 0.23	514/2616 (19.6)	1.22 (1.04–1.44); 0.01	1283/3280 (39.1)	1.07 (0.90–1.27); 0.45
Vocational center	72/190 (37.9)	1.40 (1.24–1.57); <0.001	1/1 (100%)	–	11/46 (23.9)	1.02 (0.86–1.20); 0.83	69/184 (37.5)	1.06 (0.88–1.27); 0.53
TB contact in 2 years	768/2508 (30.6)	1.35 (1.22–1.50); <0.001	155/739 (21.0)	1.39 (1.13–1.71); 0.002	573/2012 (28.5)	1.45 (1.26–1.66); <0.001	301/775 (38.8)	1.09 (0.99–1.21); 0.09
No contact in 2 years	3468/16,061 (21.6)	Reference	1108/8903 (12.5)	Reference	1578/9131 (17.3)	Reference	1874/4702 (39.9)	Reference
Received BCG	4091/18,096 (22.6)	1.13 (0.92–1.40); 0.25	1258/9574 (13.1)	0.77 (0.59–1.00); 0.053	2112/10,914 (19.4)	1.17 (0.93–1.48); 0.17	2058/5190 (39.6)	1.25 (0.97–1.61); 0.08
No BCG	101/347 (29.1)	Reference	8/52 (15.4)	Reference	37/203 (18.2)	Reference	72/189 (38.1)	Reference
Hepatitis B	149/397 (37.5)	1.12 (0.98–1.27); 0.09	20/76 (26.3)	1.99 (1.30–3.02); 0.001	48/184 (26.1)	1.18 (0.96–1.44); 0.11	114/255 (44.7)	1.06 (0.85–1.31); 0.37
No hepatitis B	4098/18,184 (22.5)	Reference	1251/9575 (13.1)	Reference	2107/10,964 (19.2)	Reference	2063/5224 (39.5)	Reference
Smoking^d^	–	–	–	–	–	–	64/146 (43.8)	1.03 (0.88–1.23); 0.66
No smoking							2113/5333 (39.6)	Reference
Diabetes mellitus	–	–	–	–	–	–	35/59 (59.3)	0.94 (0.74–1.19); 0.60
No diabetes							2142/5420 (39.5)	Reference

a TBI was calculated using data from all participants who received tuberculin skin test. ‘N’ represents distinct number of individuals.

b Adjusted prevalence ratio: log-binomial regression models are adjusted for age, sex, year, residential institute, TB exposure, BCG vaccine, and hepatitis B, as appropriate.

c The count is greater than the total no. of participants because of multiple episodes of screening per participant. Institutes that had received ≥2 rounds of screening were included.

d Ever smoked cigarette vs never smoked cigarette.

**Table 3:** Risk factors associated with acquisition of TB infection (TST conversion) in children, adolescents, and adults in congregate settings (2017–2024).

Risk factor	All participants		Children (5–14 years)		Adolescents (10–19 years)		Adults (≥18 years)	
	TST conversion^a^ (N = 5515), n (%)	aPR^b^ (95% CI); p value	TST conversion^a^ (N = 4213), n (%)	aPR^b^ (95% CI); p value	TST conversion (N = 3359), n (%)	aPR^b^ (95% CI); p value	TST conversion (N = 649), n (%)	aPR^b^ (95% CI); p value
Age, median (IQR)	15 (13–18)	1.02 (1.01–1.03); 0.001	12 (11–13)	0.96 (0.91–1.02); 0.18	14 (12–16)	0.97 (0.92–1.02); 0.21	27 (20–39)	1.02 (1.01–1.03); <0.001
Males	640/4107 (15.6)	1.34 (1.18–1.53); <0.001	263/2125 (12.4)	1.15 (1.02–1.29); 0.02	488/3422 (14.3)	1.37 (1.25–1.5); <0.001	187/734 (25.5)	1.86 (1.41–2.45); <0.001
Female	486/4238 (11.5)		228/2176 (10.5)	Reference	350/3537 (9.9)	Reference	124/747 (16.6)	Reference
Weight (kg), mean (SD)	51.5 (15.3)	1.00 (0.99–1.01); 0.56	41 (13)	1.00 (0.99–1.01); 0.79	49 (13)	1.01 (0.99–1.01); 0.11	64 (12)	1.00 (1.00–1.01); 0.30
**Year of screening**								
2018	49/266 (18.4)	Reference	20/89 (22.5)	Reference	48/249 (19.3)	Reference	–	–
2019	299/2845 (10.5)	0.59 (0.39–0.89); 0.01	175/1899 (9.2)	0.46 (0.30–0.71); 0.001	230/2365 (9.7)	0.56 (0.36–0.87); 0.01	51/267 (19.1)	Reference
2022	273/1865 (14.6)	0.70 (0.45–1.11); 0.13	114/939 (12.1)	0.63 (0.38–1.03); 0.07	200/1603 (12.5)	0.71 (0.45–1.12); 0.14	87/316 (27.5)	1.30 (0.95–1.77); 0.10
2023	231/1865 (15.0)	0.62 (0.40–0.95); 0.03	80/603 (13.3)	0.61 (0.39–0.94); 0.025	161/1291 (12.5)	0.62 (0.41–0.93); 0.02	92/419 (22.0)	1.02 (0.75–1.39); 0.90
2024	274/1828 (15.0)	0.67 (0.48–0.93); 0.02	102/771 (13.2)	0.65 (0.45–0.94); 0.02	199/1451 (13.7)	0.71 (0.47–1.08); 0.11	78/443 (17.6)	0.84 (0.61–1.17); 0.31
**Residential institute**								
School	857/7303 (11.7)	Reference	470/4203 (11.2)	Reference	744/6595 (11.8)	Reference	104/692 (15.0)	Reference
Monastery/nunnery	263/999 (26.3)	1.66 (1.23–2.25); 0.001	21/98/73 (21.4)	1.70 (1.13–2.58); 0.01	93/351 (26.5)	2.26 (1.66–3.08); <0.001	201/749 (26.8)	1.48 (1.16–1.88); 0.001
TB contact in 2 years	224/1446 (15.5)	1.17 (0.99–1.39); 0.07	60/438 (13.7)	1.24 (0.88–1.74); 0.21	178/1246 (14.3)	1.30 (1.04–1.60); 0.02	68/335 (20.3)	1.11 (0.82–1.51); 0.51
No contact in 2 years	891/6886 (12.9)	Reference	422/3852 (11.0)	Reference	651/5702 (11.4)	Reference	243/1146 (21.2)	Reference
Received BCG	1100/8200 (13.4)	0.88 (0.65–1.18); 0.39	485/4275 (11.4)	–	829/6888 (12.0)	0.90 (0.49–1.65); 0.73	295/1395 (21.2)	–
No BCG	9/55 (16.4)	Reference	5/20 (25)		6/44 (13.6)	Reference	2/16 (12.5)	
Hepatitis B	27/139 (19.4)	1.11 (0.85–1.45); 0.42	5/37 (13.5)	1.44 (0.77–2.69); 0.26	10/16 (10.4)	0.96 (0.44–2.07); 0.91	114/255 (44.7)	1.37 (1.00–1.88); 0.049
No hepatitis B	1099/8206 (13.4)	Reference	486/4264 (11.4)	Reference	828/6863 (12.1)	Reference	2063/5224 (39.5)	Reference

a The risk of TST conversion was calculated among participants with longitudinal follow up who were TST negative at baseline. ‘N’ represents distinct number of persons for whom TST conversion results are available. However, the counts for the risk factors are generated from longitudinal data and therefore, the denominators do not add up to 'N'.

b Adjusted prevalence ratio: log-binomial regression models are adjusted for age, sex, year, residential setting, TB exposure, BCG vaccine, and hepatitis B, as appropriate.

### Reduction in prevalence and risk of TBI

**Fig. 3:**
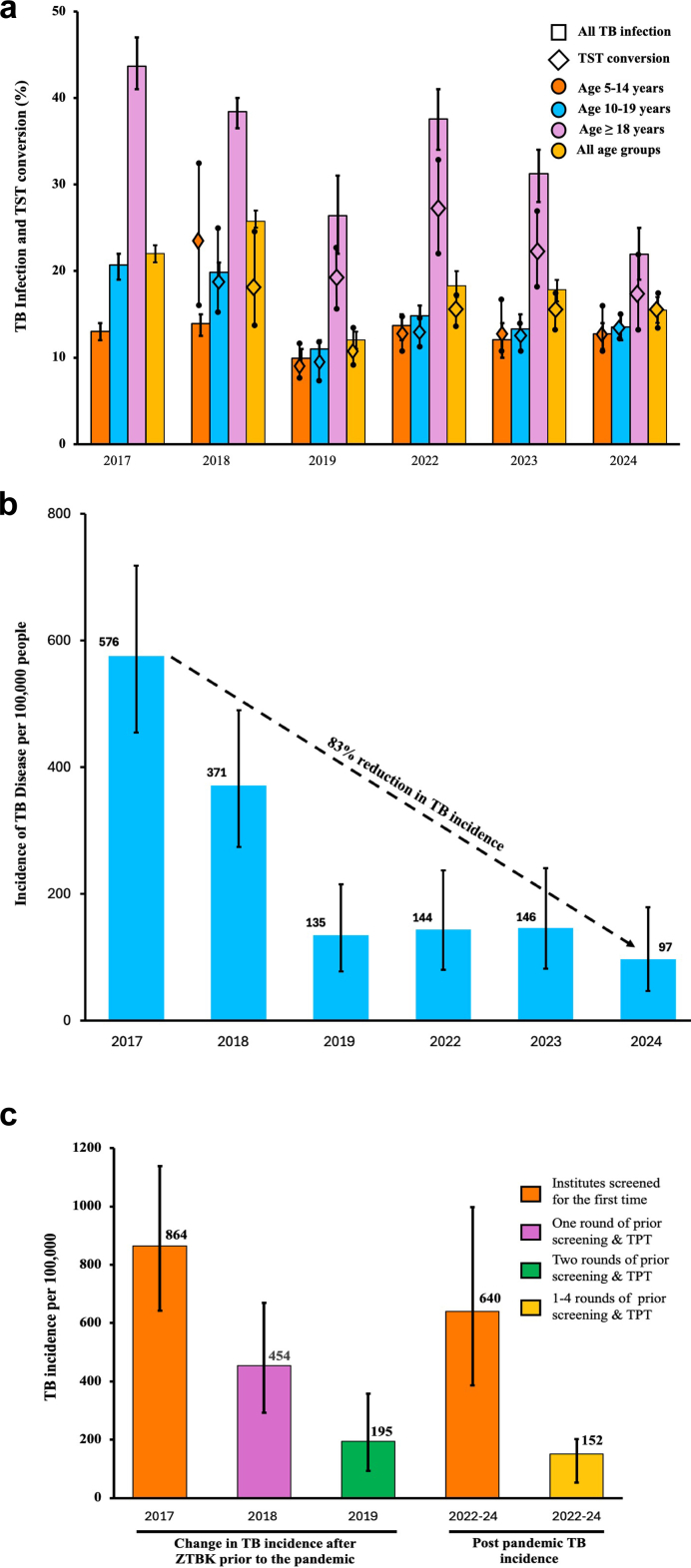
**Impact of widescale TB screening and TPT on reduction of TB burden in congregate settings in India.** a) Prevalence of TBI and Proportion of TST conversion in all age groups between 2017 and 2024. TBI is represented by bar graphs which is color coded age group wise, diamond represent TST conversion. Data was lacking for the determination of TST conversion for adults in 2018. b) TB disease incidence between 2017 and 2024. Each bar represents TB incidence per 100,000 population. TB screening could not be carried out in 2020 and 2021 due to the pandemic. c) TB incidence between 2017 and 2018 after one round of screening and TPT and after two rounds of screening and TPT between 2017 and 2019. Error bars in the panels represent 95% confidence intervals.

The prevalence of TBI in the institutes that had previously received at least one round of screening and TPT decreased 32% between 2017 and 2024, from 22% (n = 1247/5257) to 15% (n = 481/3110) (Table 2, Table S1). Before the pandemic between 2017 and 2019, the prevalence declined by 45%, from 22% (n = 1247/5657) to 12% (n = 452/3757) (Fig. 3a, Table S2). A rebound in the prevalence of TBI and TST conversion was observed after the pandemic in 2022–2024 (Table 3, Table S2). The prevalence of TBI in the 22 institutes that were screened for the first time after the pandemic was 28% (n = 594/2116).

### TB disease and TPT

Of the 20,068 schoolchildren and adults screened, TB disease were diagnosed in 121 participants, 98 schoolchildren ≤19 years of age, and 23 adults ≥20 years of age (Table S3). 7% of schoolchildren and 22% of adults with TB disease had rifampin- or multidrug-resistant TB. The prevalence of asymptomatic TB was 37% in schoolchildren and 55% in adults. The prevalence of TB with no cough were 52% in schoolchildren and 70% in adults. Among people with TBI, the proportion of TB disease was 0.08% (n = 4/4880) in those who received TPT and 4.4% (n = 95/2144) in those who did not. Followed up over eight years, children and adolescents who received TPT had a five-fold lower prevalence and 82% lower risk of TB disease (aHR: 0.18; 95% CI: 0.06–0.48) than those who did not receive TPT (Table S4, Fig. S1).

### Reduction in TB incidence and herd effect

**Fig. 4:**
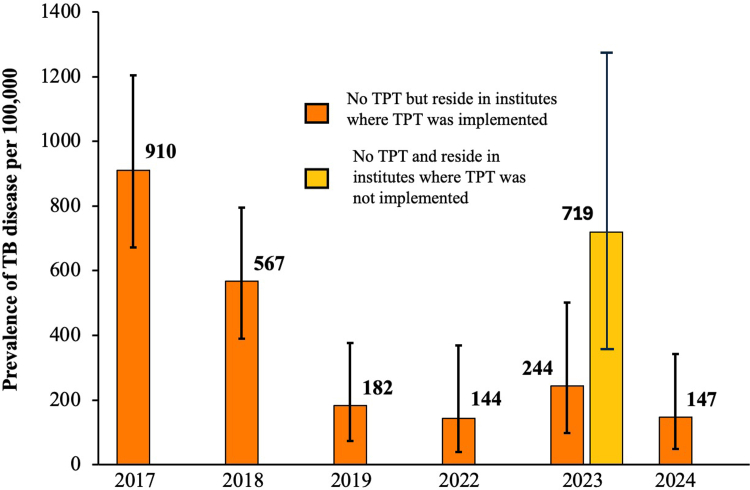
**Herd effect of TPT implementation.** TB incidence among participants who did not receive TPT but reside in facilities where TPT was implemented. Error bars represent 95% confidence intervals.

For institutes that received 1–4 prior rounds of TB screening and TPT implementation by 2024, the incidence of TB disease decreased 83%, from 576 (95% CI: 455–718) in 2017 to 97/100,000 (95% CI: 47–179) in 2024 (Table S1, Fig. 3b). For institutes that received one prior round, the incidence decreased 47% [864 (95% CI: 642–1138) in 2017 to 454 (95% CI: 293–669) in 2018] (Fig. 3c). In participants who did not receive TPT, the prevalence of TB disease decreased 84%, from 910 (95% CI: 675–1204) in 2017 to 147 (95% CI: 48–343) in 2024 (Fig. 4). TB incidence in the institutes that had not received prior screening and TPT implementation was 640/100,000 (95% CI: 386–997) in 2022–24.

### TPT uptake, completion and side-effects

Overall, 84% (n = 2820/3304) of the eligible children and adolescents and 51% (n = 1020/2005) of the eligible adults received TPT (Table S5). Increasing age was associated with greater uptake in schoolchildren (aPR: 1.01; 95% CI: 0.99–1.01) and lower uptake in adults [0.98 (0.97–0.99)]. Adult males were more likely to receive TPT than females [53% vs 47%; aPR: 1.12 (1.00–1.25)]. Hepatitis B [43% vs 72.6%; aPR: 0.71 (0.6–0.84)] and seizure disorder were associated with lower uptake of TPT [24% vs 72% (aPR: 0.31 (0.15–0.65)]. The completion of TPT was high for both schoolchildren (96%) and adults (87%), and similar for 3HR and 4R (Table S6). The frequency of hepatotoxicity was low for adolescents (4R: 0.2%; 3HR: 1.7%) and adults (4R: 0.4%; 3HR: 3.2%) and none for children (Table S7).

## Discussion

We have previously described 87% reduction in TB disease in boarding schools over three years in a cohort of 7389 participants.^7^ In an expanded cohort of 20,068 participants representing 67,637 person-years from more diverse setting, we now demonstrate a sustained reduction of incidence of TB by 83% and prevalence of TBI by 32% between 2017 and 2024, despite setbacks by the COVID-19 pandemic. Analogous to the herd immunity in vaccination campaigns, we observed significant reduction of TB among participants who did not receive TPT. Single round of screening and TPT had led to 47% reduction in TB incidence and 40–60% reduction in TST conversion. The TB resurgence that was observed after the pandemic was likely due to the disruption or diversion of health services and lockdowns during the pandemic that halted TB screening and TPT services. The significant reduction of TST conversion as well as the herd benefit among participants that didn't receive TPT are evidence of the program's impact on the reduction of TB transmission.

Our observation of 82% TB risk reduction in children and adolescents who received TPT is similar to the 79% risk reduction we observed previously.^5^ Independent clinical trials had shown TB disease protection from isoniazid preventive treatment (IPT) between 60 and 67% in children less than 15 years of age.^4^^,^^9^, ^10^, ^11^, ^12^, ^13^ One clinical trial had evaluated the efficacy of three months of daily rifampin (3R) or 3HR as TPT against placebo in patients with silicosis.^14^ In this, 52% and 41% protection against TB disease was observed for 3R and 3HR, respectively. In healthy patients, the efficacy of four months of rifampin was found to be non-inferior to 9-month IPT.^15^ Our observation of 82% protection against TB disease for TPT recipients may be attributable to a greater benefit of TPT in younger age population in whom TBI may have been mostly recently acquired and to high completion.

Applying the TB incidence of 576/100,000 observed in the initial year in 2017 to the average annual population in the congregate settings of 12,000, 553 individuals (69 annually) could have been affected by TB over the eight years in the absence of ZTBK as opposed to 121 people with TB. As such, 432 students and staff were likely prevented from developing TB disease without inclusion of the secondary disease development from potential onward transmission. Applying similar logic, several thousand TB infections were prevented. Although the long-term benefit of TPT was debated in the past largely as a result of negative findings from studies carried out among gold miners in South Africa,^16^^,^^17^ we demonstrated benefit of TPT in sustained reduction of TB burden.

Although several studies have described higher risk of TBI^18^, ^19^, ^20^ and TB disease^21^, ^22^, ^23^ in male adults, evidence for similarly higher risk in children and adolescents is limited. Few studies have reported higher^19^ or null^24^^,^^25^ risk of TBI in male school-aged children, in relatively small samples. We have previously observed a 33% greater risk of TBI in male children and adolescents.^5^ We continue to observe similar risk for males across age groups in a decisive sample size. Traditional risk factors such as smoking, alcohol use, and outdoor activity^20^^,^^21^^,^^26^^,^^27^ that were linked to the greater risk of TB in males are inapplicable in these settings. Moreover, less TB exposure was recorded for males across the age groups. These observations may indicate the role of underlying biological mechanisms, such as those related to sex hormones and immune response,^28^, ^29^, ^30^ for the greater risk of TBI among males. However, we did not find greater risk of TB disease in males. While this may be due to the limited number of TB disease episodes, biological mechanisms driving sex-based difference in acquisition of TBI vs TB progression must be further investigated.

The 50–60% greater risk of TBI and three-fold greater risk of TB disease progression in recently exposed children and adolescents indicate that ongoing transmission is driving TB in children and adolescents, rather than reactivation of remote infection. We did not observe such increased risk of TBI for similarly exposed adults. The proportion of drug resistance was also high, 7% in children and adolescents and 22% in adults, compared to 3.2% of incident DR-TB patients globally.^1^ These factors, along with the high prevalence of subclinical disease that we observed previously as well,^5^ highlight the importance of active screening, detection of drug resistance, and appropriate treatment. We observed that the reported prevalence of HBV infection across the age groups were high even though the community has been covered under universal immunization program that includes hepatitis B vaccination. Factors driving the transmission of HBV in the community must be studied. We also observed a greater prevalence and risk of TBI among the children with HBV. The few studies that had investigated the association between HBV and TBI appear to corroborate our findings.^31^^,^^32^ A relative immunosuppressed state has been described in persons with HBV, with increased CD4+CD25+ FOXP3+ regulatory T-cell and IL-10 levels, and diminished IFN-γ responses.^33^, ^34^, ^35^ Immediately, we did not observe noticeable social or environmental factors predisposing participants with hepatitis B to greater TB exposure. We observed that of the 176 schoolchildren and adults with chronic hepatitis B, only 43% received TPT, and none developed hepatotoxicity. However, people with HBV infection who received TPT likely constituted the healthier subset of patients with normal liver enzymes, ultrasound reports, and viral load, and were mostly not receiving antiviral medications. Therefore, TPT tolerance among people with HBV described here may not be generalizable to those with compromised liver function or more advanced disease. The lower uptake of TPT among participants with hepatitis B was likely due to the perceptions of harm of TPT, however unsubstantiated, to liver by participants and parents, and possibly providers. The significantly lower uptake of TPT among schoolchildren and adults with seizure were likely due to concerns or uncertainty among providers regarding drug–drug interactions between TPT and anti-epileptic agents, and related dose adjustments. We recommend initiating a call to action to develop guidelines for managing TBI and TPT implementation in patients with seizure and hepatitis B to avoid disadvantaging subgroups who may be at greater risk of TB progression. TPT was generally well-tolerated, there was no difference in the frequency of side-effects between 3HR and 4R regimens in children <10 years of age. For adolescents and adults, 3HR was more frequently associated with side-effects than 4R. These findings can guide age-based TPT implementation strategies. An organized awareness campaigns at schools engaging school leaders and teachers and engagement of school nurses and mothers at home in TPT supervision may have contributed to greater uptake and completion of TPT in schools than in monasteries and nunneries.

Broad guidelines for global TB elimination by 2035 exist in the World Health Organization's End TB Strategy roadmap and implementation documents.^36^, ^37^, ^38^ However, a detailed framework and case studies showing how to actually reach the set milestones such as the 95% reduction in incidence by 2035 are lacking. The End TB Strategy is also premised on the expectation that there will be breakthroughs in vaccines, treatment, or point-of-care diagnostics. While these stipulations may be applicable to global progress, our results highlight the potential of locally driven solutions tailored to communities to exceed expectations through good public health practice, using existing tools. Even one-time TB screening and TPT prior to the pandemic had substantial impact on TB burden in the following year. Tuberculosis infection is a dynamic continuum of disease spectrum that reversibly progresses between latency and clinical states, which underscores the importance of surveilling TBI and TPT as part of the global strategy to end TB, particularly for children and adolescents. Such a surveillance system would capture rising TBI and forestall disease outbreaks through TPT particularly in high burden settings.

Resource constraints and logistical challenges prevented us from prospectively defining evaluation and control areas. However, a natural experimental setting that allowed for simultaneous comparisons of TB rates between congregate facilities based on the receipt of prior intervention, at different frequencies, helped us to partly overcome this limitation. The fact that TB rates in the post-pandemic period were substantially different between institutes that did and did not receive the screening and TPT showed that the reduction in the TB incidence was attributable to the program, and not due to secular time trends or indirect effects of pandemic related social distancing or masking.^7^ Additionally, the timing of the COVID-19 pandemic roughly halfway through the project demonstrates the resilience of such a comprehensive endeavor despite sudden, unexpected interruptions emphasizing the inherent value of this approach. Other challenges faced in implementing the project pertain to sensitization of the community to latent infection and preventive treatment, which were new concepts for the community. Community mobilization engaging spiritual, political and community leaders, medical officers, as well as the school administrations helped us to overcome the challenges and successfully implement this program. While we note that the program required sufficient resources, the benefits exceeded the resources and effort expended. Nevertheless, we recommend a proper cost-effectiveness analysis for implementing such a comprehensive program.

ZTBK presents a two-part framework for TB elimination in schools and other congregate settings with high TB burden: an initial rapid-reduction phase through comprehensive screening and treatment of latent and active TB, followed by annual screening and treatment of new students who join the community. The frequency of initial comprehensive screening may be informed by the disease burden in the community. Nations and programs must make a concerted effort to scale up screening of TBI and implementation of TPT to achieve global TB elimination goals.

## Contributors

Conceptualization: KD, REC, ZP and TDS. Study design: KD, RKS, TDS, and REC. KD defined the methodology and wrote the first draft of the manuscript. KD, ST, TY, and SD curated the data. KD, SB and SD conducted the analysis. KD, REC, and ZP acquired the funding. KD, ST, RKS, TN, RK, UG, TT1, TT2, TY, TK1, TK2, TD1, TD2, TW, DL, LT, JK, and TDS conducted the investigation. TDS, KD and ST led the project administration. KD, ST, TN, RK, UG, LT, AG, RCB, JEG, SS, ZP, REC and VM provided resources. KD and RCS designed the figures. KD, TDS, RCS, SD, SB, AG, BB, and JEG reviewed and edited the manuscript. KD, TY, ST, SD and RCS accessed and verified the data. All authors had full access to all the data in the study and accept responsibility for the decision to submit for publication.

## Data sharing statement

All relevant data supporting the findings of this study are included in the main text, figures, and Supplementary Material. Requests for the following information must be approved by an independent review committee and should be directed at the corresponding author: de-identified individual participant data that underlie the results reported in this article. The scheduled terms of access will be reviewed and communicated on a case-by-case basis.

## Editor note

The Lancet Group takes a neutral position with respect to territorial claims in published maps and institutional affiliations.

## Declaration of interests

The authors have no competing interests to declare.
