# Supplementary material for: Comprehensive tuberculosis screening and preventive treatment in schools and congregate settings of India (2017–2024): a prospective study
Source: Lancet Reg Health Southeast Asia. 2026 Feb 6;46:100725. doi: 10.1016/j.lansea.2026.100725 (PMC12907724; doi:10.1016/j.lansea.2026.100725)
Supplement: Supplementary Material [file mmc1.docx]

**Supplementary Material**

[Supplementary Figures 2](#_Toc212635186)

S1 Figure. Prevalence of TBI (S1A) and disease (S1B) by age in male and female schoolchildren (2017-2024)…2

[S2 Figure. Effectiveness of tuberculosis preventive treatment in children and adolescents (2017-2024) 2](#_Toc212635188)

[Supplementary Tables 3](#_Toc212635189)

[S1 Table. Incidence and prevalence of TB in all particiapnts in schools, monasteries, and nunneries in Northern India after comprehensive TB screening and TB preventive treatment between 2017 and 2024…………………..3](#_Toc212635190)

[S2 Table. Prevalence of TBI and TST conversion in children, adolescents and adults between 2017 and 2024 in congregate settings in India…………………………………………………………………………………………3](#_Toc212635191)

[S3 Table. Characteristics of TB disease in children, adolescents and adults between 2017 and 2024 in congregate settings in India 4](#_Toc212635191)

[S4 Table. Risk factors for TB disease and effectiveness of TB preventive treatment in children and adolescents in the congregate settings in India (2017-2024) 5](#_Toc212635192)

[S5 Table. Factors associated with uptake of TB preventive treatment (TPT) in children, adolescents and adults in the schools, monasteries and nunneries in India (2017-2024) 6](#_Toc212635193)

[S6 Table. TB preventive treatment completion among schoolchildren and staff who received either 3 months of isoniazid and rifampin or 4 months of rifampin (2017-2024) 6](#_Toc212635194)

[S7 Table. Side-effects associated with TPT regimens consisting of 3HR (3-months of daily isoniazid and rifampin) or 4R (4-months of daily rifampin) in schoolchildren and adults (2017-2024) 7](#_Toc212635195)


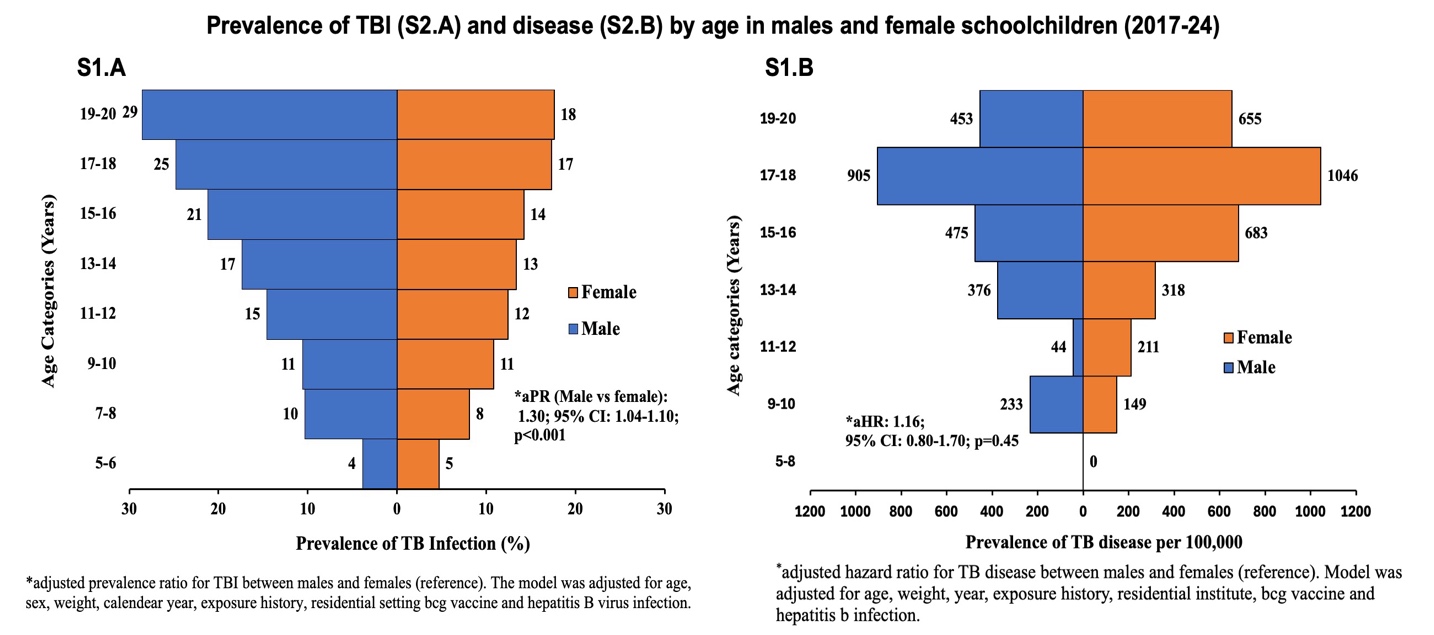


## S2 Figure. Effectiveness of tuberculosis preventive treatment in children and adolescents (2017-2024)


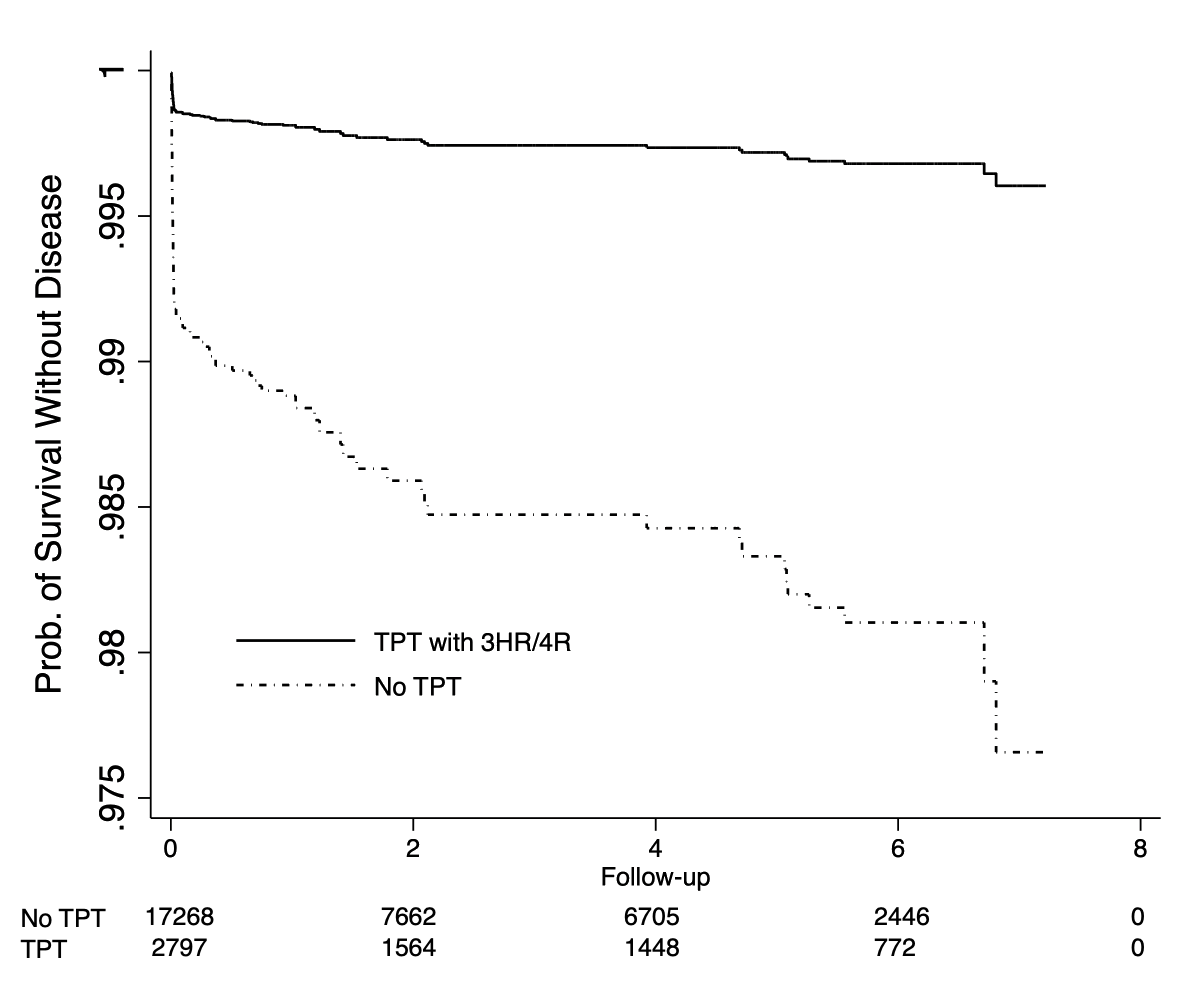


The Cox model was adjusted is adjusted for age, sex, calendar year, ethnicity, residential institute, recent contact, hepatitis B virus infection and BCG vaccine.

**Supplementary Tables**

## S1 Table. Incidence and prevalence of TB in schools, monasteries, and nunneries in the Tibetan community in Northern India after comprehensive TB screening and TB preventive treatment between 2017 and 2024

|  | **TB disease** | | | | **TB Infection** | | **TST Conversion** | |
| --- | --- | --- | --- | --- | --- | --- | --- | --- |
| **^1^Year** | **TB disease (n/N)** | **^2^Incidence per 100,000 (95% CI)** | **Annual change** | **Cumulative change** | **TB infection, n/N** | **^3^Prevalence,**  **% (95% CI)** | **TST conversion, n/N** | **Proportion,**  **% (95% CI)** |
| 2017^4^ | 78/13552 | 576 (455-718) | ·· | Reference | 1247/5657 | 22 (21-23) | .. | .. |
| 2018 | 49/13213 | 371 (274-490) | 36% ↓ | 36% ↓ | 1607/6235 | 26 (25-27) | 49/266 | 18 (14-24) |
| 2019 | 17/12630 | 135 (78-215) | 64% ↓ | 77% ↓ | 452/3757 | 12 (11-13) | 299/2845 | 11 (9-12) |
| 2022 | 15/10427 | 144 (80-237) | 7% ↑ | 75% ↓ | 577/3152 | 18 (17-20) | 273/1865 | 15 (13-16) |
| 2023 | 15/10262 | 146 (81-240) | 1% ↑ | 75% ↓ | 552/3089 | 18 (16.5-19) | 231/1541 | 15 (13-17) |
| 2024 | 10/10274 | 97 (47-179) | 33% ↓ | 83% ↓ | 481/3110 | 15 (14-17) | 274/1828 | 15 (13-17) |

^1^ All the institutes received at least two rounds of TB screening and TPT implementation.

^2^ All active TB cases detected in the students and staff in the institutes in the given year.

^3^ Prevalence of TBI in students and staff screened for TBI in the given year.

^4^ One child had TB in 2017 with a recurrent episode in 2018. Both the episodes were included as incident cases in 2017 and 2018.

## S2 Table. TB infection and TST conversion stratified by age groups in schools, monasteries, and nunneries in the Tibetan community in Northern India after comprehensive TB screening and TB preventive treatment between 2017 and 2024

|  | **Children (5-14 years)** | | | | **Adolescents (10-19 years)** | | | | **Adults (≥18 years)** | | | |
| --- | --- | --- | --- | --- | --- | --- | --- | --- | --- | --- | --- | --- |
|  | **TB Infection** | | **TST conversion** | | **TB Infection** | | **TST conversion** | | **TB Infection** | | **TST conversion** | |
| **^1^Year** | **n/N** | **% (95% CI)** | **n/N** | **%**  **(95% CI)** | **n/N** | **% (95% CI)** | **n/N** | **% (95% CI)** | **n/N** | **% (95% CI)** | **n/N** | **% (95% CI)** |
| 2017 | 434/3337 | 13 (12-14) | .. | .. | 817/3952 | 21 (19-22) | .. | .. | 453/1038 | 44 (41-47) | .. | .. |
| 2018 | 325/2332 | 14 (12.5-15) | 20/89 | 22 (14-32.5) | 698/3518 | 20 (18.5-21) | 48/249 | 19 (15-25) | 1007/2622 | 38 (36.5-40) | .. | .. |
| 2019 | 251/2525 | 10 (9-11) | 175/1899 | 9 (8-11) | 315/2863 | 11 (10-12) | 230/2365 | 10 (9-11) | 103/390 | 26 (22-31) | 51/267 | 19 (15-24) |
| 2022 | 240/1749 | 14 (12-15) | 114/939 | 12 (10-14) | 340/2292 | 15 (13-16) | 200/1603 | 12 (11-14) | 239/636 | 38 (34-41) | 87/316 | 28 (23-33) |
| 2023 | 169/1401 | 12 (10-14) | 80/603 | 13 (11-16) | 291/2187 | 13 (12-15) | 161/1291 | 12 (11-14) | 286/916 | 31 (28-34) | 92/419 | 22 (18-26) |
| 2024 | 216/1693 | 13 (11-14) | 102/771 | 13 (11-16) | 296/2191 | 14 (12-15) | 199/1451 | 14 (12-16) | 149/676 | 22 (19-25) | 78/443 | 18 (14-21.5) |

^1^ All the institutes received at least two rounds of TB screening and TPT implementation.

## S3 Table. Characteristics of TB disease in children, adolescents and adults between

## 2017 and 2024 in congregate settings in India

| ^1^**Results** | **Children and adolescents 5-19 years old with TB disease, n/N (%)** | **Adults ≥20 years with TB disease, n/N (%)** |  |
| --- | --- | --- | --- |
| **Disease site**  Pulmonary only  Extrapulmonary only | 93/98 (95%)  5/98 (5%) | 23/23 (100%)  0/23 (0%) |  |
| ^2^**Smear positive**  **Smear negative** | 16/90 (18)  74/90 (82) | 5/20 (25)  15/20 (75) |  |
| ^3^**Xpert result**  Positive  Negative | 87/96 (91)  8/96 (9) | 20/23 (87)  3/23 (13) |  |
| **Xpert *M. tb* load**  Trace  Very low  Low  Medium  High | 3/87 (3.5)  27/87 (31)  35/87 (40)  20/87 (23)  2/87 (2) | 1/20 (5)  7/20 (35)  7/20 (35)  5/20 (25)  0/20 (0) |  |
| **Rifampin resistance (Xpert)**  Sensitive  Resistant | 82/87 (94)  5/87 (6) | 16/20 (80)  4/20 (20) |  |
| ^4^**Culture result**  Positive  Negative | 37/85 (43.5)  48/85 (56.5) | 8/17 (47)  9/17 (53) |  |
| **Drug resistance**  Multi-drug resistant-TB  Non MDR-TB  Isoniazid mono-resistant | 7/98 (7)  91/98 (93)  2/98 (2) | 5/23 (22)  18/23 (78)  1/23 (4) |  |
| **TB related symptoms**  Any Cough  No cough  ^5^Asymptomatic TB | 44/98 (45%)  54/98 (55%)  36/98 (37%) | 7/23 (30%)  16/23 (70%)  12/23 (52%) |  |
| ^1^Note that the total number of TB cases includes TB cases that were detected during the ZTBK screening and not passively. Therefore, the total number of TB cases are different from that of Table 3 that additionally includes cases detected passively outside the screening.  ^2^Smear microscopy was not performed for 11 cases.  ^3^Xpert was not performed for two extrapulmonary TB cases  ^4^Culture was performed on Xpert positive samples. Eight Xpert positives did not get culture or results were missing.  ^5^No cough, hemoptysis, fever, night sweats, tiredness, weight loss, or chest pain. | | | |

## S4 Table. Risk factors for TB disease and effectiveness of TB preventive treatment

## In children and adolescents in the congregate settings in India (2017-2024)

| **Risk Factor** | ^1^**Prevalence of TB, n/N (per 100,000)** | **Unadjusted HR  (95% CI); p-value** | **^2^aHR (95% CI);**  **p-value** |  |
| --- | --- | --- | --- | --- |
| **TB Preventive Treatment**  ^3^Received TPT  Did not receive TPT | 4/2819 (140)  93/12272 (760) | 0.26 (0.15-0.45); <0.001  Reference | 0.18 (0.10-0.30); <0.001  Reference |  |
| Age, median (IQR) | 16 (14-17) | 1.28 (1.19-1.39); <0.001 | 1.31 (1.20-1.44); <0.001 |  |
| ^4^Males  Female | 50/8747 (570)  48/6345 (760) | 0.73 (0.52-1.03); 0.08  Reference | 1.16 (0.79-1.70); 0.45  Reference |  |
| Weight in kg, mean (SD) | 51 (12.4) | 1.04 (1.02-1.05); <0.001 | 0.99 (0.97-1.02); 0.48 |  |
| ^4^**Calendar year**  2017  2018  2019  2022  2023  2024 | 45/5291 (850)  28/4587 (610)  5/4310 (120)  5/3374 (150)  10/4016 (250)  4/3286 (120) | Reference  0.67 (0.27-1.65); 39  0.26 (0.08-0.82); 0.02  0.26 (0.11-0.50); 0.002  0.42 (0.22-0.82); 0.01  0.40 (0.19-0.85); 0.02 | Reference  0.91 (0.30-2.74); 0.86  0.29 (0.07-1.25); 0.10  0.44 (0.19-1.01); 0.60  0.76 (0.38-1.53); 0.44  0.99 (0.53-1.87); 0.99 |  |
| **Residential institute**  School  Monastery and nunnery | 91/12000 (760)  7/3043 (230) | Reference  0.25 (0.10-0.61); 0.002 | Reference  0.22 (0.08-0.67); 0.007 |  |
| TB contact in 2 years  No contact in 2 years | 44/12221 (360)  49/2860 (1710) | 6.47 (4.03-10.39); <0.001  Reference | 3.37 (2.02-5.59); <0.001 |  |
| ^4^Received BCG  No BCG | 93/24475 (380)  2/271 (740) | 0.61 (0.23-1.60); 0.31  Reference | 2.2 (0.76-6.41); 0.14  Reference |  |
| Hepatitis B  No hepatitis B | 2/207 (970)  96/14885 (640) | 1.93 (0.42-8.91); 0.40  Reference | 0.88 (0.20-3.90); 0.86  Reference |  |
| ^1^TB disease detected outside the screening were not included, as risk factor information was not available for them.  ^2^The model is adjusted for age, sex, calendar year, residential institute, recent contact, hepatitis B virus infection and BCG vaccine.  ^3^One person with Xpert “Trace” result and culture negative was put on TPT and not included.  ^4^Because of longitudinal data, the denominators do not add up to match the denominator for other risk factors. | | | | |

## S5 Table. Factors associated with uptake of TB preventive treatment (TPT) in children, adolescents and adults in the schools, monasteries and nunneries in India (2017-2024)

| **Participant characteristic or risk factor** | **All participants** | | **Children and adolescents**  **5-19 years** | | **Adults≥18 years** | |
| --- | --- | --- | --- | --- | --- | --- |
|  | **Received TPT/ Eligible for TPT,**  **n/N (%)** | **aPR^*^ (95% CI); p-value** | **Received TPT/ Eligible for TPT, n/N (%)** | **aPR^*^ (95% CI);**  **p-value** | **Received TPT/ Eligible for TPT, n/N (%)** | **aPR^*^ (95% CI); p-value** |
| Received TPT/ Eligible for TPT | 3841/5359 (71.7) | .. | 2820/3304 (84) | ·· | 1020/2005 (50.9) | ·· |
| Age, median (IQR) | 16 (13-20) | 0.98 (0.97-0.99); <0.001 | 14 (12-16) | 1.01 (0.99-1.01); 0.05 | 31 (24-41) | 0.98 (0.97-0.99); <0.001 |
| Males  Females | 2432/3357 (72.5)  1408/2002 (70.3) | 1.04 (1.0-1.08); 0.01  Reference | 1758/2090 (84)  1062/1264 (84) | 1.01 (0.97-1.05); 0.71  Reference | 674/1267 (53.2)  346/738 (46.9) | 1.12 (1.00-1.25); 0.04  Reference |
| **Institute type**  School  Monastery/nunnery  Vocational center | 2636/3320 (79.4)  1143/1967 (58.1)  61/72 (84.7) | Reference  0.87 (0.74-1.03); 0.12  1.11 (0.96-1.17); 0.22 | 2320/2658 (87.3)  490/685 (71.5)  10/11 (90.9) | Reference  0.82 (0.70-0.02); 0.02  1.0 (0.99-1.09); 0.11 | 316/662 (47.7)  653/1282 (50.9)  51/61 (83.6) | Reference  0.92 (0.72-1.19); 0.54  1.16 (1.04-1.29); 0.007 |
| Recent TB exposure  No recent exposure | 708/872 (81.2)  3125/4476 (69.8) | 1.09 (1.04-1.14); 0.12  Reference | 577/670 (86.1)  2236/2675 (83.6) | 1.00 (0.96-1.05); 0.91  Reference | 131/202 (64.9)  889/1801 (49.4) | 1.20 (1.05-1.36); 0.006  Reference |
| TST size, mean (SE) | 16.3 (5.5) | 1.003 (0.99-1.01); 0.25 | 16 (5) | 1.01 (0.96-1.05); 0.06 | 18 (6.5) | 1.003 (99-1.01); 0.43 |
| Smoker  Non-smoker | .. | .. | ·· | ·· | 28/59 (47.5)  992/1946 (51) | 0.91 (0.77-1.07); 0.26  Reference |
| Hypertension  No hypertension | .. | .. | ·· | ·· | 43/97 (44.3)  970/1900 (51.1) | 1.08 (0.85-1.35); 0.48  Reference |
| Diabetes  No diabetes | .. | .. | ·· | ·· | 24/43 (55.8)  996/1899 (52) | 1.24 (0.93-1.64); 0.14  Reference |
| Hepatitis B  No hepatitis B | 75/176 (42.6)  3765/5183 (72.6) | 0.71 (0.6-0.84); <0.001  Reference | 46/62 (74.2)  2774/3292 (84.3) | 0.85 (0.72-1.01); 0.07  Reference | 29/114 (25.4)  991/1891 (52.4) | 0.63 (0.50-0.80); <0.001  Reference |
| Acid peptic disease  No acid peptic disease | 34/68 (50.0)  3806/5291 (71.9) | 0.93 (0.75-1.15); 0.49  Reference | 11/15 (73.3)  2809/3339 (84.1) | 0.99 (0.84-1.17); 0.91  Reference | 23/53 (43.4)  997/1952 (50.9) | 0.91 (0.67-1.22); 0.51  Reference |
| Seizure disorder  No seizure | 6/25 (24)  3834/5334 (71.9) | 0.31 (0.15-0.65); 0.002  Reference | 5/17 (29.4)  2815/3337 (84.4) | 0.26 (0.1-0.66); 0.005  Reference | 1/8 (12.5)  1019/1997 (51.0) | 0.45 (0.20-1.01); 0.05  Reference |
| ^*^The model is adjusted for age, sex, type of institute, recent exposure, history of smoking, and the presence of diabetes mellitus, hepatitis B, acid peptic disease and seizure. | | | | | | |

## S6 Table. TB preventive treatment completion among schoolchildren and staff who received either

## three months of isoniazid and rifampin or four months of rifampin (2017-2024)

| **TPT Regimen** | **TPT Outcome^*^** | **All participants (N=3537), n (%)** | **Children and adolescents (N=2623), n (%)** | **Adults**  **(N=914), n (%)** |
| --- | --- | --- | --- | --- |
| All TPT | TPT completed  ^@^TPT stopped prematurely  Loss to follow  Self-discontinued  Other | 3297/3537 (93.2)  5/3537 (0.14)  34/3537 (1)  181/3537 (5.1)  20/3537 (0.6) | 2502/2623 (95.4)  4/2623 (0.2)  18/2623 (0.7)  89/2623 (3.4)  10/2623 (0.4) | 795/914 (87)  1/914 (0.1)  16/914 (1.8)  92/914 (10.1)  10/914 (1.1) |
| 3HR | TPT completed  TPT stopped prematurely  Loss to follow  Self-discontinued  Other | 886/900 (98.4)  5/900 (0.6)  6/900 (0.7)  2/900 (0.2)  1/900 (0.1) | 762/773 (98.6)  4/773 (0.5)  5/773 (0.7)  1/773 (0.1)  1/773 (0.1) | 124/127 (97.6)  1/127 (0.8)  1/127 (0.8)  1/127 (0.1)  ·· |
| 4R | TPT completed  TPT stopped prematurely  Loss to follow  Self-discontinued  Other | 2408/2631 (91.5)  ··  26/2631 (1)  179 /2631 (6.8)  18/2631 (0.7) | 1738/1845 (94.2)  ··  11/1845 (0.6)  86/1845 (4.8)  8/1845 (0.4) | 670/786 (85.2)  ··  15/786 (1.9)  91/786 (11.6)  10/786 (1.3) |
| ^*^TPT was ongoing for 310 participants, 160 students and 150 staff.  ^@^Instances where TPT was prematurely stopped by a clinician due to toxicities. | | | | |

## S7 Table. Side-effects associated with TPT regimens consisting of 3HR (3-months of daily isoniazid and rifampin) or 4R (4-months of daily rifampin) in schoolchildren and adults (2017-2024)

| **Side-effect** | **Children (5-9 years)** | | | | **Adolescents (10-19 years)** | | | **Adults (≥20 years)** | | |
| --- | --- | --- | --- | --- | --- | --- | --- | --- | --- | --- |
|  | **3HR,**  **n (%)** | **4R,**  **n (%)** | ***χ^2^***  **p value** | **3HR,**  **n (%)** | | **4R,**  **n (%)** | ***χ^2^***  **p value** | **3HR,**  **n (%)** | **4R,**  **n (%)** | ***χ^2^***  **p value** |
| Gastrointestinal^1^ | 3/65 (4.6) | 2/191 (1.1) | 0.07 | 51/708 (7.2) | | 32/1838 (1.7) | <0.001 | 10/127 (7.9) | 60/948 (6.4) | 0.52 |
| Central Nervous System^2^ | 2/65 (3.1) | 3/191 (1.6) | 0.45 | 97/708 (13.7) | | 40/1838 (2.2) | <0.001 | 18/127 (14.2) | 51/948 (5.4) | <0.001 |
| Rash/Acne | 1/65 (1.5) | 1/191 (0.5) | 0.42 | 24/708 (3.4) | | 1/1838 (0.05) | <0.001 | 1/127 (0.8) | 13/948 (1.4) | 0.58 |
| Hepatotoxicity | 0/65 (0) | 0/191 (0) | ·· | 12/708 (1.7) | | 3/1838 (0.2) | <0.001 | 4/127 (3.2) | 4/948 (0.4) | 0.001 |
| Tiredness | 0/65 (0) | 1/191 (0.5) | 0.56 | 58/708 (8.2) | | 17/1838 (0.9) | <0.001 | 17/127 (13.4) | 21/948 (2.2) | <0.001 |
| Any side effect | 6/65 (9.2) | 5/191 (2.6) | 0.023 | 184/708 (26) | | 77/1838 (4.2) | <0.001 | 35/127 (27.6) | 117/948 (12.3) | <0.001 |
| Serious adverse events | 0/65 (0) | 0/167 (0) | ·· | 0/708 (0) | | 0/1838 (0) | ·· | 0/127 (0) | 0/936 (0) | ·· |
| ^1^Gastrointestinal side-effects: nausea, vomiting, gastritis, heartburn, decreased appetite, diarrhea.  ^2^Central nervous systems side-effects: headache, dizziness, sleepiness, forgetfulness. | | | | | | | | | | |
